# The impact of thrombocytopenia on variceal bleeding in cirrhotic patients with transjugular intrahepatic portosystemic shunt

**DOI:** 10.1038/s41598-023-28646-5

**Published:** 2023-01-30

**Authors:** Yang Chen, Chongtu Yang, Songjiang Huang, Jiacheng Liu, Yingliang Wang, Chen Zhou, Tongqiang Li, Chaoyang Wang, Shuguang Ju, Yaowei Bai, Wei Yao, Bin Xiong

**Affiliations:** 1 Department of Radiology, Union Hospital, Tongji Medical College, Huazhong University of Science and Technology, No.1277 Jiefang Avenue, Wuhan, 430022 China; 2 Hubei Province Key Laboratory of Molecular Imaging, Wuhan, 430022 China

**Keywords:** Gastrointestinal bleeding, Liver diseases

## Abstract

Thrombocytopenia is the most frequent haematologic disorder in patients with cirrhosis, and it is perceived as a contributory factor for bleeding events. Cirrhosis patients with portal hypertension (PHT) is often accompanied with mild to moderate thrombocytopenia when they treated with transjugular intrahepatic portosystemic shunt (TIPS). To address whether the risk of variceal hemorrhage after TIPS varies with different platelet count in patients with normal platelet count and thrombocytopenia, we conducted the retrospective controlled study to evaluate the association of platelet count with the risk of variceal bleeding after TIPS. 304 patients were selected to the study. Propensity score matching was performed to adjust for potential selection bias. 63 patients from each group could be paired. Cox proportional hazards models were used to evaluate the association between platelet and variceal bleeding after TIPS. Platelet counts of two groups are 185.0 ± 98.7 × 10^9^/L (normal platelet count) and 70.6 ± 39.3 × 10^9^/L (thrombocytopenia) respectively. The bleeding rates of two groups in overall cohort are 10.9% (normal platelet count) and 12.9% (thrombocytopenia). After matched, the bleeding rates of two groups are 11.1% (normal platelet count) and 14.3% (thrombocytopenia) There was no statistically significant difference in bleeding rates between the two groups, either in the whole cohort (*P* = 0.671) or in the matched cohort (*P* = 0.593). Platelet count was not associated with bleeding events after TIPS (hazard ratio (HR) 95% confidence interval: 0.986–1.005, *P* = 0.397 in normal platelet count and 95% confidence interval: 0.968–1.020, *P* = 0.648 in thrombocytopenia). Thrombocytopenia in patients with cirrhosis was not associated with the risk of variceal bleeding episodes post-TIPS. Thrombocytopenia should not be viewed as an absolute contraindication for TIPS.

## Introduction

Decompensated cirrhosis is generally associated with low platelet count which has been suggested as contributing to bleeding^1^. Gastrointestinal bleeding is the second most common complication in patients with decompensated cirrhosis after ascites, and variceal bleeding is the main cause of hemorrhage events^2^. Despite improvements in management, variceal bleeding is associated with a mortality that is still approximately 15–20% at 6 weeks after onset^3,4^. Transjugular intrahepatic portosystemic shunts (TIPS) is a well-established therapy in cirrhosis patients with variceal bleeding who do not respond to pharmacologic agents or endoscopic treatment^5–7^.

TIPS creates an intrahepatic tract between the hepatic and portal vein, subsequently reducing portal hypertension and improving variceal bleeding by diverting blood into the systemic circulation and away from the portal^8^. In patients suffering from refractory or recurrent ascites, TIPS is also recommended for therapy^5^. The incidence of hepatic encephalopathy and post-operative bleeding restricts the clinical application of TIPS. Although, TIPS shows a lower rate of recurrent bleeding in comparison to drug or endoscopic therapy^4,9^. Post-variceal hemorrhage is likely to be fatal, and often results in impaired liver function, and can lead to other life-threatening complications. In addition, thrombocytopenia is a common complication of chronic liver disease, affecting 78% of cirrhotic patients^10^. Surgical splenectomy can significantly improve platelet counts in patients with cirrhosis with hypersplenism and thrombocytopenia^11,12^. Cirrhosis variceal bleeding patients without splenectomy tend to exhibit moderate to severe thrombocytopenia, when treated with TIPS.

Our center has collected a cohort of patients with retrospective cohort study based on TIPS, a portion of patients accepted splenectomy before TIPS placement to reach the normal platelet count. Meanwhile patients with no splenectomy had thrombocytopenia in the cohort. As a result, we had a unique opportunity to further explore the association between thrombocytopenia and variceal bleeding events following TIPS placement.

## Materials and methods

### Study patients

The retrospective analysis of all cirrhosis patients with PHT who were consecutively admitted to our center and received TIPS treatment from February 2016 to September 2021. The exclusion criteria included, noncirrhotic patients, necessary clinical data not known and lost to follow-up within 6 weeks after TIPS placement. Thus, patients diagnosed with cirrhosis of any etiology who underwent successful TIPS were considered eligible for the study, and they were categorized into two groups according to whether they had a previous history of splenectomy. The diagnosis of cirrhosis was based on medical history, imaging, and/or liver biopsy. Clinical characteristics, laboratory tests, and radiographic results were collected from the electronic medical record during hospitalization of the patients. Laboratory tests and clinical evaluations of post-bleeding and survival were conducted at each outpatient visit every 3 months, supplemented by telephone visits. After TIPS creation, all patients are followed-up at 1, 3, 6, and 12 months and then annually thereafter. All patients underwent follow-up until death, liver transplantation or the end of research (December, 2021). The data were censored at the end of follow-up period. The main endpoint of the study was variceal bleeding and the second was all-cause mortality.

The present observational study was conducted at Wuhan Union Hospital. The study protocol conforms to the ethical guidelines of the 1975 Declaration of Helsinki and was approved by the Wuhan Union Hospital Institutional Review Board. Informed consent was waived by the institutional review board of the Union Hospital, Tongji Medical college, Huazhong University of Science and Technology because the data have been anonymized.

### TIPS procedure

TIPS creation was performed by experienced interventional radiologists. Catheterization of the hepatic vein was implemented through the right internal jugular vein with a transjugular liver access set (RUPS-100; Cook Inc.). Then a TIPS needle was used to puncture the portal vein under fluoroscopic guidance. After successful puncture of the portal vein with a TIPS needle, a hydrophilic guidewire (Terumo, Tokyo, Japan) was sequentially introduced into the main portal vein, superior mesenteric vein or splenic vein. After the intrahepatic tract was dilated with a balloon catheter, an 8 mm ePTFE-covered stent (Fluency; Bard Corporation or Viabahn; Gore Corporation) was placed in the intrahepatic duct. Measurement of portal pressure gradient (PPG) was performed before and after shunt establishment. The target value of PPG was below 12 mmHg or, alternatively, a reduction of at least 20% from the baseline^13^. For varicose vein embolization, coil and tissue glue were used to close the varicose veins. Balloon tamponade was used when massive bleeding occurred.

### Statistical analysis

Quantitative variables are expressed as means and standard deviations and compared with Student’s t-test or Mann–Whitney test. Qualitative variables were presented as frequencies and percentages and compared by means of chi-squared test or two-tailed Fisher’s exact test.

We used a propensity score approach to control for observed confounding factors that might influence both group assignment and outcome^14^. The primary analysis was based on propensity matching. We used a 1:1 matching algorithm without replacement to match splenectomy and non- splenectomy patients on Child–Pugh score, Model for end-stage liver disease score (MELD), INR, before-PPG, post-PPG, embolization of varices and propensity score within a caliper of 0.1 standard deviation of the logit of the propensity score. The probability of post-bleeding in both groups were estimated by Kaplan–Meier curves and compared using Cox models with robust variance to account for correlations within the matched pairs^15^.

### Ethics approval and consent to participate

The study was approved by the Wuhan Union Hospital Institutional Review Board. Informed consent was waived by the institutional review board of the Union Hospital, Tongji Medical college, Huazhong University of Science and Technology because the data have been anonymized.

## Results

### Study patients

**Figure 1:**
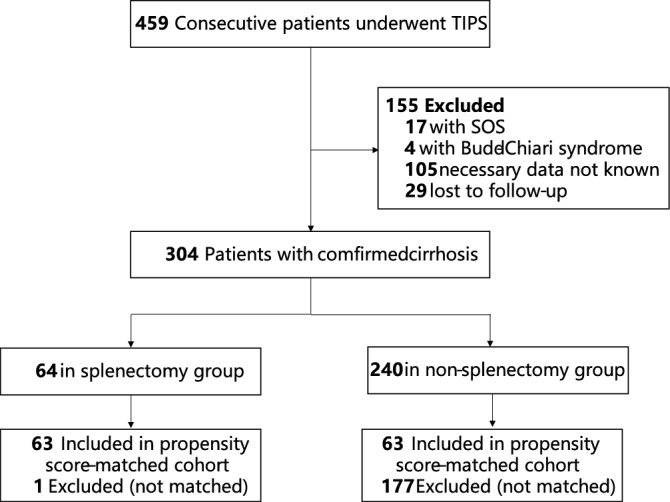
Flowchart of the patient selection protocol. SOS, hepatic sinus obstruction syndrome.

Four hundred fifty-nine consecutive cirrhosis patients with PHT who received TIPS placement at our center were retrospectively analyzed. 155 patients were excluded according to exclusion criteria. Finally, 304 patients met the inclusion criteria and were enrolled in the final study (Fig. 1). Baseline patients’ characteristics are summarized in Table 1. Propensity scores were calculated for 304 patients with confirmed cirrhosis. Among the 304 patients with confirmed cirrhosis, 126 could be matched, with 63 in each group, splenectomy and non-splenectomy. Compared with the non-splenectomy group, the splenectomy group had higher level of platelet count, lower albumin, post-PPG and MELD score. We then used a 1:1 matching algorithm without replacement to match Child–Pugh score, MELD score, INR, before-PPG, post-PPG, embolization of varices for splenectomy and non-splenectomy patients with a caliper of 0.1 of logit standard deviation propensity score. The matched groups had no difference in levels of albumin, post-PPG and MELD score. The levels of platelet count in splenectomy (181.4 ± 95.2 × 10^9^/L, normal platelet count) were in a normal range and still higher than non-splenectomy (68.4 ± 37.9 × 10^9^/L, thrombocytopenia).

**Table 1:** Select baseline characteristics.

Characteristics	All Patients	Overall cohort			Matched cohort		
		Splenectomy	Non-splenectomy	*P* value	Splenectomy	Non-splenectomy	*P* value
Age	55.2 ± 11.8	55.97 ± 10.1	55.00 ± 12.3	0.606	55.97 ± 10.2	53.62 ± 12.5	0.249
Gender(%)							
Male	198(65.1)	42(65.6)	156(65.0)	0.926	41(65.1)	38(60.3)	0.581
Female	106(34.9)	22(34.4)	84(35.0)		22(34.9)	25(39.7)	
Etiology of cirrhosis(%)							
HBV	175(57.6)	37(57.8)	138(57.6)	0.728	37(58.7)	39(61.9)	0.566
HCV	33(10.9)	9(14.0)	24(10.0)		9(14.3)	4(6.3)	
Alcohol	23(7.6)	4(6.3)	19(7.9)		4(6.3)	3(4.8)	
Autoimmune hepatitis	18(5.9)	2(3.1)	16(6.6)		2(3.2)	4(6.3)	
Others	55(18.1)	12(18.8)	43(17.9)		11(17.5)	13(20.6)	
Tips indications(%)							
Variceal bleeding	273(89.8)	61(95.3)	212(88.3)	0.101	60(95.2)	55(87.3)	0.115
Refractory ascites	31(10.2)	3(4.7)	28(11.7)		3(4.8)	8(12.7)	
Child–Pugh score	7.6 ± 1.6	7.7 ± 1.7	7.6 ± 1.6	0.438	7.7 ± 1.7	7.9 ± 1.6	0.558
Meld Score	11.6 ± 3.5	10.8 ± 3.7	11.8 ± 3.4	0.032	10.8 ± 3.7	12.1 ± 3.9	0.062
Meld-Na Score	12.5 ± 4.6	12.0 ± 5.2	12.65 ± 4.5	0.355	12.1 ± 5.3	13.0 ± 4.8	0.314
Bilirubin (μmol/L)	26.5 ± 26.1	23.1 ± 24.9	27.4 ± 26.4	0.24	23.3 ± 25.0	29.1 ± 37.9	0.312
Albumin (g/L)	30.6 ± 5.3	28.8 ± 4.9	31.1 ± 5.4	0.002	28.8 ± 5.0	30.2 ± 5.7	0.152
Hb(g/L)	80.0 ± 19.4	80.3 ± 16.5	83.9 ± 24.8	0.181	80.6 ± 16.5	79.4 ± 17.2	0.697
PT (s)	16.7 ± 2.6	16.2 ± 2.3	16.8 ± 2.7	0.142	16.3 ± 2.3	16.9 ± 2.5	0.131
Platelet count (× 10^9^/L)	94.8 ± 73.8	185.0 ± 98.7	70.6 ± 39.3	0	181.4 ± 95.2	68.4 ± 37.9	0
INR	1.4 ± 0.3	1.3 ± 0.2	1.4 ± 0.3	0.102	1.3 ± 0.2	1.4 ± 0.3	0.179
PPG (mm Hg)							
Before TIPS	27.4 ± 6.1	26.4 ± 5.2	27.7 ± 6.3	0.136	26.4 ± 5.3	26.0 ± 6.2	0.625
After TIPS	12.1 ± 4.5	10.5 ± 3.5	12.6 ± 4.7	0.001	10.5 ± 3.5	9.7 ± 3.0	0.169

### Probability of variceal bleeding after TIPS

**Figure 2:**
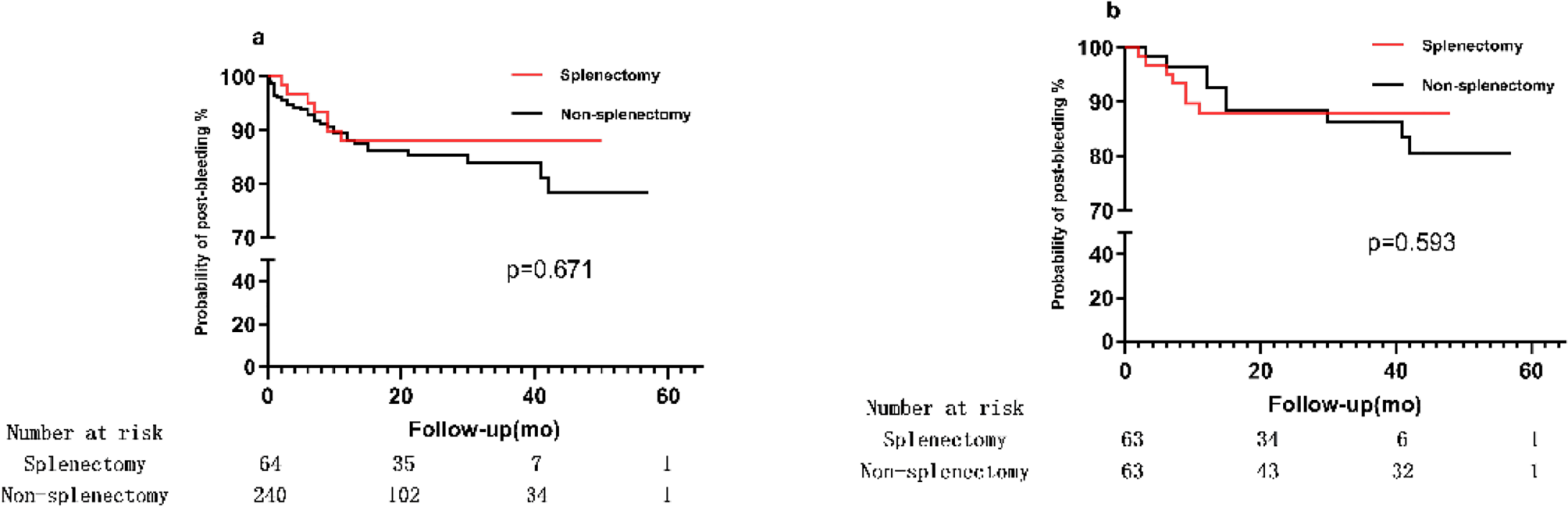
Kaplan–Meier survival curves of the splenectomy group and non-splenectomy group. (**a**) Post-bleeding Probability in the overall Cohort. (**b**) Post-bleeding Probability in the matched Cohort.

In matched cohort, 7(11.1%) patients in normal platelet count experienced postoperative variceal bleeding, and 9(14.3%) patients in thrombocytopenia recured variceal hemorrhage. There was no statistically significant difference between normal platelet count group and thrombocytopenia group (HR 95% confidence interval: 0.261–2.156, *p* = 0.593). Meanwhile, in overall cohort, 31(12.9%) patients in thrombocytopenia experienced post-bleeding, and 7(10.9%) patients in normal platelet count had postoperative variceal bleeding. Before matched, the probability of post-bleeding in normal platelet count and thrombocytopenia also had no difference (HR 95% confidence interval: 0.347–1.978, *p* = 0.671). Probability of variceal bleeding of two groups is shown in Table 2. Kaplan–Meier analysis is shown in Fig. 2.

**Table 2:** Probability of variceal bleeding after TIPS.

Outcome	Overall cohort			Matched cohort		
	Non-splenectomy	Splenectomy	HR (95% CI)	Non-splenectomy	Splenectomy	HR (95% CI)
Post-bleeding (%)	31 (12.9)	7 (10.9)	0.347–1.978	9 (14.3)	7 (11.1)	0.261–2.156

### Independent predictor of post-bleeding

The univariate and multivariable Cox regression analysis was performed in Table 3. The final model of multivariable Cox regression analysis showed that levels of platelet count was not able to predict the occurrence of hemorrhage after TIPS. In addition, Child–Pugh score, MELD score, INR pre-PPG and post-PPG were also not predictors the risk of variceal bleeding after TIPS.

**Table 3:** Univariate and multivariate analyses according to the incidence of post-bleeding during the observation period in matched group.

	Univariate analysis				Multivariate analysis			
	Splenectomy		Non-splenectomy		Splenectomy		Non-splenectomy	
	HR (95% CI)	P value	HR (95% CI)	P value	HR (95% CI)	P value	HR (95% CI)	P value
Child–Pugh score	0.643–1.710	0.850	0.487–1.236	0.285	0.254–1.162	0.116	0.269–0.921	0.026
Meld Score	0.942–1.298	0.219	0.890–1.299	0.453	0.699–1.287	0.735	0.993–1.970	0.055
INR	0.866–106.932	0.065	0.111–24.052	0.721	0.709–203,225.988	0.064	0.009–91.331	0.971
Platelet count (× 10^9^/L)	0.987–1.006	0.440	0.978–1.021	0.937	0.988–1.009	0.711	0.980–1.031	0.701
PPG (mm Hg)								
Before TIPS	0.861–1.170	0.963	0.920–1.125	0.740	0.906–1.299	0.376	0.869–1.140	0.950
After TIPS	0.792–1.239	0.933	0.853–1.290	0.650	0.760–1.272	0.897	0.838–1.380	0.567

## Discussion

The results of this retrospective study in cirrhotic patients with different platelet levels indicate that preoperative platelet count is not a predictor for variceal bleeding events following the creation of TIPS. At the same time, thrombocytopenia in patients with cirrhosis was not associated with the risk of postoperative variceal hemorrhage.

Thrombocytopenia is a general hematological disorder in patients with cirrhosis^10^, and is generally defined as any decrease in platelet counts below the lower normal limit (i.e. < 150 × 10^9^/L)^16^. The development of thrombocytopenia in cirrhosis is complex and the severity of liver disease is the major factor in the process^10^. The lifespan of a normal platelet is about10 days and one-third is sequestered in the spleen^17^. In cirrhosis patients, hypersplenism due to PHT is the most important cause of thrombocytopenia^18^. As a result, for cirrhotic patients with persistent platelet reduction, surgical splenectomy is an effective therapy to improve platelet counts in patients with PHT-induced hypersplenism^18^. In the overall cohort, 64 patients had splenectomy so that their platelet counts were within a normal range before TIPS placement. The mean (± SD) platelet count in the splenectomy (normal platelet count) group is 185.0 ± 98.7 × 10^9^/L and 70.6 ± 39.3 × 10^9^/L in the non-splenectomy (thrombocytopenia) group. There was a significant different in the overall platelet count between patients who underwent splenectomy prior to TIPS versus those who did not. It has been established that a low platelet count raises the risk of bleeding^19,20^.

Prevention and treatment of variceal hemorrhage are essential to improve the prognosis of patients with cirrhosis. TIPS is currently an effective treatment for avoiding hemorrhage in cirrhosis patients who do not to respond to pharmacological therapy and endoscopic therapy. However, its clinical application is still constrained by the risk of hepatic encephalopathy and the risk of variceal bleeding after TIPS^21^. In a previous study, splenectomy did not affect variceal bleeding after TIPS, but it may be related to the hemodynamic alterations^22^. Therefore, the purpose of\the current study, is to look at how platelet count affects postoperative variceal bleeding. According to our data, the probability of variceal bleeding after TIPS placement has no significant difference between normal platelet count and thrombocytopenia (*p* = 0.671). We used a propensity score approach to minimize biases in order to eliminate confounding factors that could affect the probability of hemorrhage. Nevertheless, even once the propensity score was matched, there was no significant difference in bleeding rate after TIPS between two groups (*p* = 0.593). A COX regression model additionally showed that platelet counts are not a risk factor for variceal bleeding following TIPS.

Our findings demonstrate a link between platelet counts and variceal bleeding post-TIPS. According to a prior study, platelet count had no impact on predicting bleeding events in patients with cirrhosis^23^. Platelet counts do not predict variceal bleeding in cirrhotic patients treated with TIPS, which we further confirmed in our research. The most likely explanation is that high PPG, not thrombocytopenia, is the primary cause of variceal bleeding^23,24^. Because post-PPG in the whole cohort had been reduced to below 12 mmHg or reduction of at least 20% from the baseline, it could explain that PPG did not correlate with variceal bleeding result in the statistical analysis^13^.

It’s interesting to note that in our study, MELD and Child–Pugh score were not significantly associated with higher bleeding rates after TIPS. A possible explanation for this finding is that, whereas these two score systems could predict mortality after TIPS treatment, they were less successful in predicting variceal post-bleeding events^25,26^. Variceal bleeding after TIPS may not be the primary cause of death in cirrhosis patients. Recent research by Bucsics et al. shown that thrombocytopenia, anemia, and leukopenia frequently improved after TIPS^27^. Thrombocytopenia should not be regarded as an absolute contraindications to TIPS.

Our study has several limitations. Although we tried to reduce biases as much as possible by using a variety of statistical approaches Uncontrolled confounding factors are the fundamental drawback of our study, as they are in all observational studies.. Additionally, we performed a propensity score analysis between normal platelet count and thrombocytopenia, leading to the small sample size. Beyond all of that, considering the relatively low bleeding rate, we cannot completely rule out that our sample is too small to assess the predictive value of platelet count. The extrapolation of results could be impacted by our study cohort’s high incidence of variceal bleeding prior TIPS. Further study is required to assess the predictive significance of platelet count for variceal bleeding after TIPS.

In conclusion, according to our results, the risk of variceal bleeding after TIPS between normal platelet counts and thrombocytopenia shows no statistically significant difference. Furthermore, platelet count levels before TIPS cannot predict the risk of variceal bleeding after TIPS. Thrombocytopenia should not be viewed as absolute contraindications for TIPS. This study provides some reference to clinicians in selecting TIPS treatment in patients with cirrhosis with thrombocytopenia.

## Data Availability

The datasets during and/or analyzed during the current study available from the corresponding author on reasonable request. The data is not publicly available to protect patient privacy.

## References

[1] Chalasani N, Imperiale TF, Ismail A, Sood G, Carey M, Wilcox CM, Madichetty H, Kwo PY, Boyer TD (1999). Predictors of large esophageal varices in patients with cirrhosis. Am. J. Gastroenterol..

[2] Ginès P, Krag A, Abraldes JG, Solà E, Fabrellas N, Kamath PS (2021). Liver cirrhosis. Lancet Lond. Engl..

[3] Hernández-Gea V, Berbel C, Baiges A, García-Pagán JC (2018). Acute variceal bleeding: Risk stratification and management (Including TIPS). Hepatol. Int..

[4] de Franchis R, Faculty Baveno VI (2015). Expanding consensus in portal hypertension: Report of the baveno VI consensus workshop: Stratifying risk and individualizing care for portal hypertension. J. Hepatol..

[5] Tripathi D, Stanley AJ, Hayes PC, Travis S, Armstrong MJ, Tsochatzis EA, Rowe IA, Roslund N, Ireland H, Lomax M, Leithead JA, Mehrzad H, Aspinall RJ, McDonagh J, Patch D (2020). Transjugular intrahepatic portosystemic stent-shunt in the management of portal hypertension. Gut.

[6] Henderson JM (1986). Variceal bleeding: Which shunt?. Gastroenterology.

[7] Liu J, Shi Q, Xiao S, Zhou C, Zhou B, Yuan F, Zheng C, Lin S, Qian K, Feng G, Xiong B (2020). Using transjugular intrahepatic portosystemic shunt as the first-line therapy in secondary prophylaxis of variceal hemorrhage. J. Gastroenterol. Hepatol..

[8] Rössle M, Haag K, Ochs A, Sellinger M, Nöldge G, Perarnau JM, Berger E, Blum U, Gabelmann A, Hauenstein K (1994). The transjugular intrahepatic portosystemic stent-shunt procedure for variceal bleeding. N. Engl. J. Med..

[9] Njei B, McCarty TR, Laine L (2017). Early transjugular intrahepatic portosystemic shunt in US patients hospitalized with acute esophageal variceal bleeding. J. Gastroenterol. Hepatol..

[10] Peck-Radosavljevic M (2017). Thrombocytopenia in chronic liver disease. Liver. Int. Off. J. Int. Assoc. Study. Liver..

[11] Anegawa G, Kawanaka H, Uehara H, Akahoshi T, Konishi K, Yoshida D, Kinjo N, Hashimoto N, Tomikawa M, Hashizume M, Maehara Y (2009). Effect of laparoscopic splenectomy on portal hypertensive gastropathy in cirrhotic patients with portal hypertension. J. Gastroenterol. Hepatol..

[12] Giannini E, Botta F, Borro P, Risso D, Romagnoli P, Fasoli A, Mele MR, Testa E, Mansi C, Savarino V, Testa R (2003). Platelet count/spleen diameter ratio: Proposal and validation of a non-invasive parameter to predict the presence of oesophageal varices in patients with liver cirrhosis. Gut.

[13] Monescillo A, Martínez-Lagares F, Ruiz-del-Arbol L, Sierra A, Guevara C, Jiménez E, Marrero JM, Buceta E, Sánchez J, Castellot A, Peñate M, Cruz A, Peña E (2004). Influence of portal hypertension and its early decompression by TIPS placement on the outcome of variceal bleeding. Hepatol. Baltim. Md.

[14] Rosenbaum PR, Rubin DB (1983). The central role of the propensity score in observational studies for causal effects. Biometrika.

[15] Liang KY, Zeger SL (1986). Longitudinal data analysis using generalized linear models. Biometrika.

[16] Giannini EG (2006). Review article: Thrombocytopenia in chronic liver disease and pharmacologic treatment options. Aliment. Pharmacol. Ther..

[17] O’Leary JG, Greenberg CS, Patton HM, Caldwell SH (2019). AGA Clinical practice update: Coagulation in cirrhosis. Gastroenterology.

[18] Afdhal N, McHutchison J, Brown R, Jacobson I, Manns M, Poordad F, Weksler B, Esteban R (2008). Thrombocytopenia associated with chronic liver disease. J. Hepatol..

[19] Uhl L, Assmann SF, Hamza TH, Harrison RW, Gernsheimer T, Slichter SJ (2017). Laboratory predictors of bleeding and the effect of platelet and RBC transfusions on bleeding outcomes in the PLADO trial. Blood.

[20] Friedmann AM, Sengul H, Lehmann H, Schwartz C, Goodman S (2002). Do basic laboratory tests or clinical observations predict bleeding in thrombocytopenic oncology patients? A reevaluation of prophylactic platelet transfusions. Transfus. Med. Rev..

[21] Chen S, Li X, Wei B, Tong H, Zhang M-G, Huang Z-Y, Cao J-W, Tang C-W (2013). Recurrent variceal bleeding and shunt patency: prospective randomized controlled trial of transjugular intrahepatic portosystemic shunt alone or combined with coronary vein embolization. Radiology.

[22] Yang C, Liu J, Shi Q, Huang S, Zhou C, Wang Y, Li T, Chen Y, Xiong B (2021). Effect of splenectomy on the outcomes in patients with cirrhosis receiving transjugular intrahepatic portosystemic shunt. J. Gastroenterol. Hepatol..

[23] Basili S, Raparelli V, Napoleone L, Talerico G, Corazza GR, Perticone F, Sacerdoti D, Andriulli A, Licata A, Pietrangelo A, Picardi A, Raimondo G, Violi F (2018). PRO-LIVER collaborators platelet count does not predict bleeding in cirrhotic patients: Results from the PRO-LIVER study. Am. J. Gastroenterol..

[24] Casado M, Bosch J, García-Pagán JC, Bru C, Bañares R, Bandi JC, Escorsell A, Rodríguez-Láiz JM, Gilabert R, Feu F, Schorlemer C, Echenagusia A, Rodés J (1998). Clinical events after transjugular intrahepatic portosystemic shunt: Correlation with hemodynamic findings. Gastroenterology.

[25] Malinchoc M, Kamath PS, Gordon FD, Peine CJ, Rank J, ter Borg PC (2000). A model to predict poor survival in patients undergoing transjugular intrahepatic portosystemic shunts. Hepatol. Baltim. Md.

[26] Conn HO (1981). A peek at the child-turcotte classification. Hepatol. Baltim. Md.

[27] Bucsics T, Lampichler K, Vierziger C, Schoder M, Wolf F, Bauer D, Simbrunner B, Hartl L, Jachs M, Scheiner B, Trauner M, Gruenberger T, Karnel F, Mandorfer M, Reiberger T (2022). Covered transjugular intrahepatic portosystemic shunt improves hypersplenism-associated cytopenia in cirrhosis. Dig. Dis. Sci..

